# Modulated switching current density and spin-orbit torques in MnGa/Ta films with inserting ferromagnetic layers

**DOI:** 10.1038/srep38375

**Published:** 2016-12-02

**Authors:** Kangkang Meng, Jun Miao, Xiaoguang Xu, Yong Wu, Jiaxing Xiao, Jianhua Zhao, Yong Jiang

**Affiliations:** 1 School of Materials Science and Engineering, University of Science and Technology Beijing, Beijing 100083, China; 2 State Key Laboratory of Superlattices and Microstructures, Institute of Semiconductors, Chinese Academy of Sciences, Beijing 100083, China

## Abstract

We report modulated switching current density and spin-orbit torques (SOT) in MnGa/Ta films with inserting very thin Co_2_FeAl and Co layers. Ferromagnetic coupling has been found in MnGa/Co_2_FeAl/Ta, resulting in a decreased effective anisotropy field. On the contrary, in MnGa/Co/Ta, antiferromagnetic coupling plays a dominant role. The switching current density ***J***_**c**_ in MnGa/Ta is 8.5 × 10^7^ A/cm^2^. After inserting 0.8-nm-thick Co_2_FeAl and Co, the***J***_**c**_ becomes 5 × 10^7^ A/cm^2^ and 9 × 10^7^ A/cm^2^, respectively. By performing adiabatic harmonic Hall voltage measurements, it is demonstrated that the inserted Co_2_FeAl layer has mainly enhanced the field-like torques, while in MnGa/Co/Ta the damping-like torques have been enhanced. Finally, the enhanced spin Hall effect (SHE) has also been studied using the spin Hall magnetoresistance measurement. The modulated ***J***_**c**_ and SOT are ascribed to the combination of magnetic coupling, Rashba effect and SHE at the interfaces.

Spin-orbit torques (SOT) effect has been demonstrated as a promising technique to control the magnetization in heavy metal (HM)/ferromagnetic metal (FM) heterostructures^1^,^2^,^3^,^4^,^5^,^6^,^7^,^8^. An in-plane electric current applied to the heterostructures with large spin–orbit coupling (SOC) and structural inversion asymmetry gives rise to the torques, which induces magnetization switching under an external magnetic field collinear with the current. The torques acting on the magnetization can be represented by so-called effective magnetic fields generated by the spin Hall effect (SHE) and the Rashba effect^9^,^10^,^11^,^12^,^13^,^14^,^15^,^16^,^17^,^18^,^19^,^20^. When a FM contacts with a HM with strong SOC such as Ta or Pt, the charge current in the HM will be converted into pure spin current due to SHE, such spin current can diffuse into the FM layer and exert torques on its magnetization, which is similar to spin transfer torque. On the other hand, spin accumulation can also take place at the FM/HM interface via the Rashba effect, which has also generated a significant effective field that causes current-induced domain nucleation and fast domain wall (DW) motion. To evaluate the size and direction of such torques or effective fields, a number of methods have been employed. Recently, Kim *et al*. have examined the measurement of adiabatic (low-frequency) harmonic Hall voltage to study the effective fields^5^. They derived an analytical formula for the harmonic Hall voltages and found that the effective fields strongly depend on the thicknesses of Ta and CoFeB layers in Ta/CoFeB/MgO heterostructures.

For practical use of the SOT in spintronics devices, it is of great importance to elucidate the factors determining the threshold switching current density ***J***_**c**_. The critical current density originates from the SHE is given by 
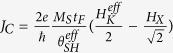
, where *H*_*X*_ is an external field collinear with the current, *e* the elementary charge, *ħ* the Dirac constant, and 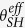 the effective spin Hall angle. *M*_*S*_, *t*_*F*_ and 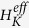 are the saturation magnetization, thickness and effective anisotropy field of the ferromagnetic layer, respectively^21^. The critical current density for the Rashba effect is given by 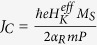, where *M*_*S*_ is the saturation magnetization, *α*_*R*_ the Rashba constant, *P* the electron spin polarization, and *m* the electron mass^22^. In a word, the switching current density is essentially determined by the characteristics of the HM and FM in the heterostructures. New paradigms for decreasing the switching current density will be possible by engineering the magnetic properties of the FM and HM/FM interfaces. On the other hand, there are two kinds of direct exchange coupling at the interface of two FM leads: ferromagnetic and antiferromagnetic. Therefore, it provides a convenient way to modulate the magnetic properties and switching current density.

The perpendicular magnetic anisotropy (PMA) of the thin ferromagnetic films used for most of the reported SOT measurements stems from the interfacial effect^2^,^3^,^4^,^5^. Zhao *et al*. have also investigated the SOT induced magnetization switching in Ta/TbFeCo structures with bulk PMA^8^. Recently, we have systematically investigated the anomalous Hall effect (AHE) in Mn_1.5_Ga/Ta bilayers, in which the PMA originates from bulk rather than interface, and the SOT induced magnetization switching has also been observed^23^. As an attempt to modulate the switching current density ***J***_**c**_ and study the underlying physics, we further investigate the magnetic properties and SOT in MnGa/Ta films with inserting Co_2_FeAl and Co films. Recent works have proved that the magnetic coupling in MnGa/Co_2_FeAl and MnGa/Co is different, which provides a way to investigate the influence of ferromagnetic and antiferromagnetic coupling on the switching current density^24^,^25^. On the other hand, we select Co_2_FeAl and Co as inserting layers because they have similar saturation magnetization of 1100 emu/cm^3^and are just designed to induce different anisotropy field^26^,^27^. Ferromagnetic coupling has been found in MnGa/Co_2_FeAl/Ta, resulting in a decreased effective anisotropy field. On the contrary, in MnGa/Co/Ta, antiferromagnetic coupling plays a dominant role. It is found that the ***J***_**c**_ in MnGa/Ta is about 8.5 × 10^7^ A/cm^2^. After inserting a 0.8-nm-thick Co_2_FeAl, ***J***_**c**_ decreases to 5 × 10^7^ A/cm^2^. However in MnGa/Co/Ta the value is increased and even larger than that in MnGa/Ta. By performing adiabatic harmonic Hall voltage measurements, we show that the inserted Co_2_FeAl layer has enhanced the effective fields, especially the field-like effective field, while the Co layer has mainly enhanced the damping-like field. The larger ***J***_**c**_ in MnGa/Co/Ta is ascribed to its larger anisotropy field. Furthermore, the modulated SHE has also been studied using the spin Hall magnetoresistance (SMR) measurements.

## Results

### Device structure and current distributions

**Figure 1:**
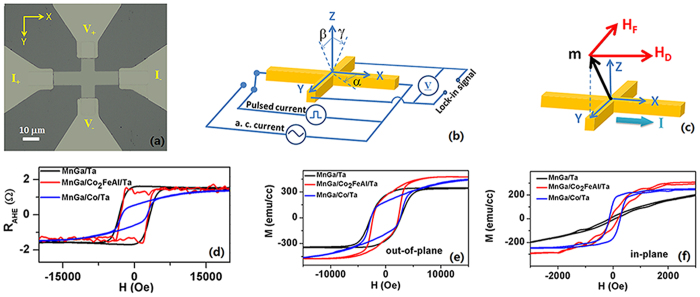
Device structure and magnetic properties. (**a**) SEM image of a patterned Hall bar. (**b**) Schematic measurement setup along with the definition of the coordinate system. (**c**) The corresponding field-like effective field **H**_**F**_ and damping-like effective field **H**_**D**_ when the magnetization is tilted perpendicular to the current direction. (**d**) Hysteresis loops of anomalous Hall resistance for the three samples. (**e**) Out-of-plane and (**f**) in-plane **M-H** curves of the three samples.

The as-prepared samples are denoted as MnGa/Ta, MnGa/Co_2_FeAl/Ta, MnGa/Co/Ta and MnGa/Co/Al respectively and the Mn/Ga atomic ratio is 1^28^. A scanning electron microscope (SEM) image of a patterned Hall bar is shown in Fig. 1a. The size of all the Hall bars is 10 μm × 80 μm. The two electrodes for current injection are labelled **I**_**+**_ and **I**_**−**_. The other two electrodes for the Hall voltage measurements are labelled **V**_**+**_ and **V**_**−**_. To evaluate the perpendicular component of the magnetization and the planar Hall effect (PHE). the Hall resistance **R**_**H**_ is measured with applying a direct current (DC) of 1 mA, corresponding to a current density of around **j **= 1 × 10^6^ A/cm^2^. Figure 1b shows the schematic of the measurement setup along with the definition of the coordinate system used in this study. We measure the SOT induced magnetization switching by applying a pulsed current with the width 50 μs, and the resistance is measured after a 16 μs delay under an external magnetic field **H**_**X**_ along either positive or negative **X** directions. We apply a sinusoidal alternating current (AC) with the amplitude of 2.1 mA and the frequency of 158.89 Hz to exert periodic SOT on the magnetization, and the first ***V***_**ω**_ and the second ***V***_**2ω**_ harmonic anomalous Hall voltages are measured as functions of magnetic field **H** at the same time using two lock-in amplifier systems. Figure 1c shows the corresponding field-like effective field **H**_**F**_ and damping-like field **H**_**D**_ when the magnetization is tilted perpendicular to the current direction.

### Magnetic properties

The hysteresis loops of the AHE for MnGa/Ta, MnGa/Co_2_FeAl/Ta, MnGa/Co/Ta are presented in Fig. 1d, and the anomalous Hall resistance **R**_**AHE**_ in all the samples are obtained by subtracting the ordinary Hall component determined from a linear fit to the high-field region up to ±6 T. We find that MnGa/Ta and MnGa/Co_2_FeAl/Ta have similar PMA properties, which has also been investigated using **M-H** measurement. Figure 1e and f show the out-of-plane and in-plane **M-H** curves of the three samples, respectively. It can be found that the saturation magnetizations of both MnGa/Co/Ta and MnGa/Co_2_FeAl/Ta are similar with each other but larger than that of MnGa/Ta. On the other hand, the out-of-plane **M-H** curve of MnGa/Ta in Fig. 1e is broad and not rectangle, indicating an in-plane component of magnetization at zero-field but the value is very small as shown in Fig. 1f. MnGa/Co_2_FeAl/Ta shows a large saturation magnetization at high out-of-plane field, but its remnant magnetization is almost the same as that of MnGa/Ta, which could be ascribed to the ferromagnetic coupling between MnGa and Co_2_FeAl^24^. On the contrary, MnGa/Co/Ta shows a small remnant magnetization and the large linear-increase of magnetization with increasing field, which indicates the antiferromagnetic coupling at the interface^25^. Both MnGa/Co_2_FeAl/Ta and MnGa/Co/Ta have non-negligible in-plane components of magnetization as shown in Fig. 1f. The effective anisotropy fields of MnGa/Ta, MnGa/Co_2_FeAl/Ta and MnGa/Co/Ta are calculated to be 1.5 T, 1.2 T and 1.7 T respectively using the reported method^12^.

### Current induced switching under H_X_ fields

**Figure 2:**
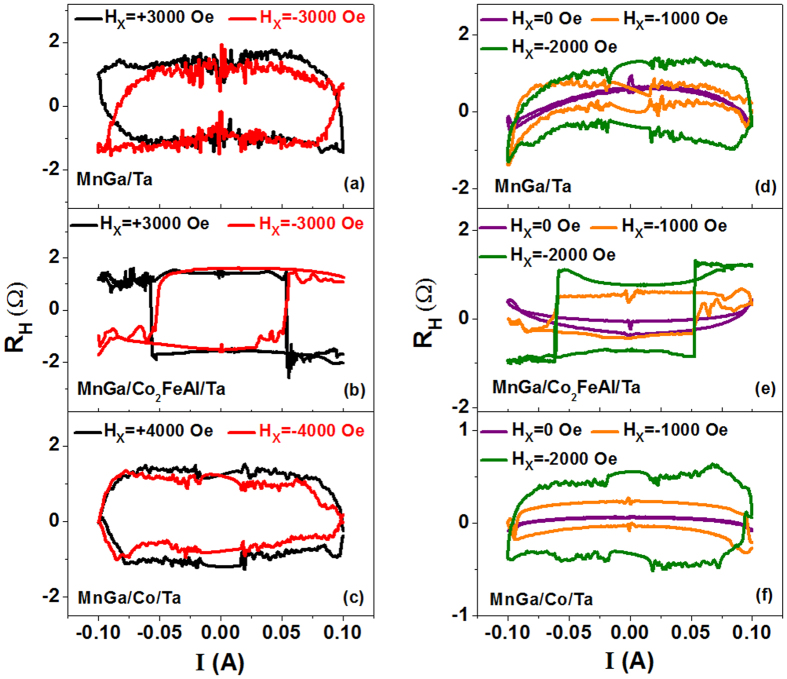
Current switching under in-plane magnetic field. **R**_**H**_-**I** curves of (**a**) MnGa/Ta and (**b**) MnGa/Co_2_FeAl/Ta with **H**_**X**_** **= ±3000 Oe. (**c**) **R**_**H**_**-I** curves of MnGa/Co_2_FeAl/Ta with **H**_**X**_** **= ±4000 Oe. (**d**–**f**) **R**_**H**_**-I** curves of the three samples with different negative **H**_**X**_.

The current-induced switching in MnGa/Ta and MnGa/Co_2_FeAl/Ta with an in-plane field of **H**_**X**_** **= ±3000 Oe are shown in Fig. 2a and b, respectively. It is found that the maximum Hall resistances of the two samples are all detected at the field. The magnetization is switched from **+Z** to **−Z** with **H**_**X**_** **= +3000 Oe when sweeping the current from negative to positive, and switched back from **−Z** to **+Z** when sweeping the current reversely. With **H**_**X**_** **= −3000 Oe, the opposite switching behavior is observed. The switching current density ***J***_**c**_ in MnGa/Ta is 8.5 × 10^7^ A/cm^2^. After inserting a 0.8-nm-thick Co_2_FeAl, it decreases to 5 × 10^7^ A/cm^2^. Similar measurements are performed in MnGa/Co/Ta with the in-plane field of **H**_**X**_** **= ±4000 Oe, since the maximum Hall resistance is detected at this field. The ***J***_**c**_ in MnGa/Co/Ta is increased to be about 9 × 10^7^ A/cm^2^. The current switching measurements are also performed over a range of in-plane external fields as shown in Fig. 2d,e. It is found that the magnetizations of the three samples are not fully switched and the anomalous Hall resistance **R**_**H**_ becomes smaller as decreasing the field, indicating that the deterministic switching gradually vanishes, which was also observed in Pt/CoNiCo/Pt symmetric devices^29^. On the other hand, the switching behaviors of MnGa/Co/Ta with +**H**_**X**_ and −**H**_**X**_ seem to be similar. It indicates that different switching schemes happen in the sample and make the interpretation difficult, because there is evident in-plane magnetization in MnGa/Co/Ta as shown in Fig. 1f and the SOT direction may also depend on the direction of magnetization^30^. In general, the results suggest that it is easier to switch the magnetization of MnGa/Co_2_FeAl/Ta as compared with MnGa/Ta but becomes hard for MnGa/Co/Ta.

### Effective fields generated by the current

To determine the strength of the spin-orbit effective fields with inserting Co_2_FeAl and Co layers, we have performed non-resonant magnetization-tilting measurements by applying a small amplitude low frequency alternating current through the device and simultaneously sweeping a static in-plane magnetic field parallel or perpendicular to the current direction (**H**_**X**_ and **H**_**Y**_)^4^,^5^. The damping-like **H**_**D**_ and field-like effective fields **H**_**F**_ can be calculated by


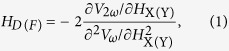


**Figure 3:**
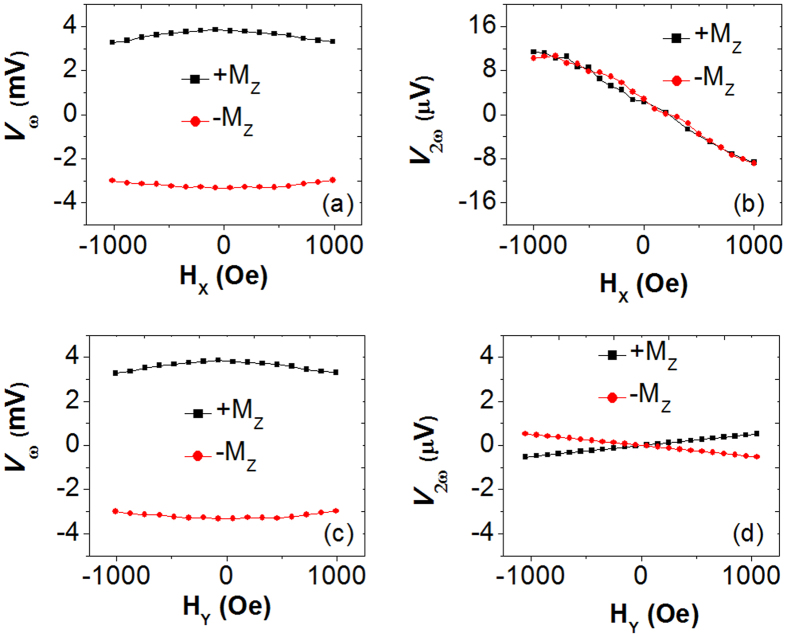
The harmonic results of the MnGa/Ta bilayers. (**a**) and (**b**) The first harmonic Hall voltages ***V***_**ω**_ and the second harmonic Hall voltages ***V***_**2ω**_ plotted against the in-plane external field **H**_**X.**_ (**c**) and (**d**) The first harmonic Hall voltages ***V***_**ω**_ and the second harmonic Hall voltages ***V***_**2ω**_ plotted against the in-plane external field **H**_**Y.**_

**Figure 4:**
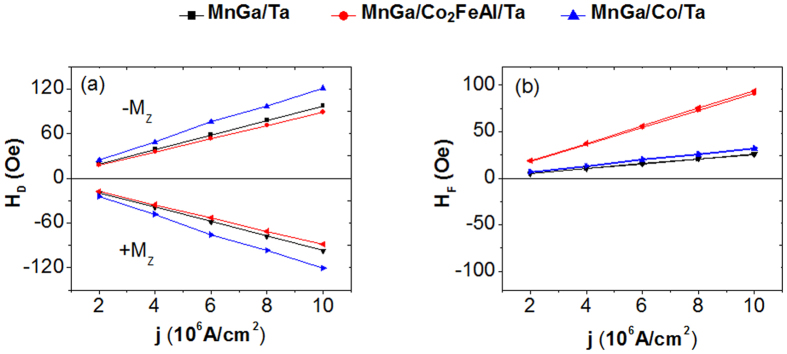
The effective fields deduced from harmonic results for the three samples.

A diagram of the measurement is shown in Fig. 1b, where the AC current with the amplitude of 2 mA was applied along the **X** axis with the external field along **X** (**H**_**X**_, α = 0°) and **Y** (**H**_**Y**_, α = 90°) axis. Figure 3 shows the harmonic results of MnGa/Ta as an example. Figure 3a and b show the first harmonic Hall voltages ***V***_**ω**_ and second harmonic Hall voltages ***V***_**2ω**_ plotted against the in-plane external field **H**_**X**_, measured with the out-of-plane magnetization component **M**_**Z**_ > 0 and with **M**_**Z**_ < 0. Figure 3c and d show the corresponding results for **H**_**Y**_. Then, the damping-like effective field **H**_**D**_ and field-like effective field **H**_**F**_ as functions of applied current density **j** are shown in Fig. 4. The effective fields vary linearly with **j**, indicating that the effects of Joule heating are negligible in the measured **j** range. The sign of **H**_**D**_ depends on the direction of **M**_**Z**_, while that of **H**_**F**_ is independent of **M**_**Z**_, which is consistent with previous reports using the same method^4^,^5^. It is indicated that the inserting the Co layer has mainly enhanced the dampling-like torques while the Co_2_FeAl layer has mainly enhanced the field-like torques.

### Spin Hall magnetoresistance

**Figure 5:**
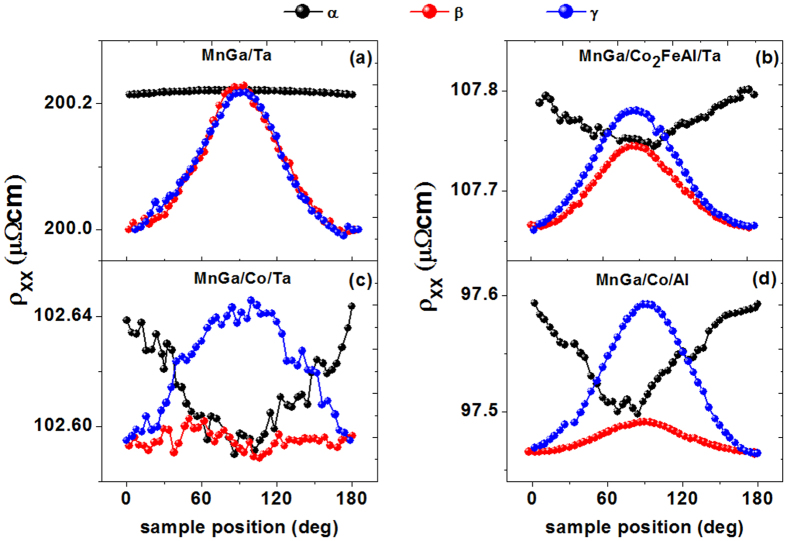
Angle-dependent longitudinal resistivity ρ_XX_ in three geometries for the four samples.

To further investigate the modulated SHE in these samples, we have carried out the SMR measurements^31^,^32^. The multilayer of MnGa/Co/Al with weak SOC was fabricated as a comparison. The SMR longitudinal resistivity change can be formulated as 
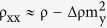
, where ρ is a constant resistivity offset, Δρ the magnitude of the resistivity changes as a function of the magnetization orientation, and **m**_**y**_ the component of the magnetization in the **Y** direction that is perpendicular to the current direction in the film plane. Therefore, the SMR effect is only related to **m**_**y**_, distinct from the ordinary anisotropic magnetoresistance (AMR) effect in magnetic layers, which depends on the **m**_**x**_ component parallel to the current direction^31^,^32^,^33^. Figure 5 shows the angle-dependent longitudinal resistivity ρ_XX_ in three geometries for the four samples, and the applied field is 9 T. It is found that the ρ_XX_ (β) and ρ_XX_ (γ) of MnGa/Ta adapt sin^4^ dependence on the angles as shown in Fig. 5a, which is caused by the AMR of MnGa as investigated in our previous work^23^. After inserting Co_2_FeAl layer, the ρ_XX_ (γ) also adapts sin^4^ dependence on the angles but the variation becomes smaller, while the ρ_XX_ (α) adapts sin^2^ dependence on the angles with a larger variation. For MnGa/Co/Ta, there is no obvious change on the ρ_XX_ (β) as compared with that in MnGa/Co/Al, where the SOC is weak. Here we will focus on the changes happened in ρ_XX_ (β), which indicate a different dependence on **m**_**y**_ and reveal the existence of SMR. We define the MR ratio as (ρ_XX_ (β = 90°)-ρ_XX_ (β = 0°))/ρ_XX_ (β = 0°). The MR of MnGa/Co/Ta is simply considered as the superposition of AMR and SMR, and the value is almost zero. The MR of MnGa/Co/Al is 0.026%, thus the SMR of MnGa/Co/Ta is about −0.026%. Recently, Kim *et al*. have studied the SMR in metallic HM/FM bilayers^34^. The SMR of a HM/FM bilayer reads


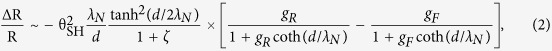



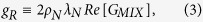



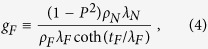


where *ρ*_*N*_, *λ*_*N*_, and θ_SH_ represent the resistivity, spin diffusion length, and spin Hall angle of the HM layer, respectively. *G*_*MIX*_ is the so-called spin mixing conductance. *t*_*F*_, *ρ*_*F*_, *λ*_*F*_, and *P* represent the thickness, resistivity, spin diffusion length, and current spin polarization of the magnetic layer, respectively. The value of *ζ* ≡ (*ρ*_*N*_*t*_*F*_*/ρ*_*F*_*d*) describes the current shunting effect into the magnetic layer. We have extracted the effective spin Hall angle θ_SH_** **= −0.11 of Ta in MnGa/Ta using the relationship H_L_** **= *ħ*θ_SH_|j|/(2|*e*|M_S_t_F_), where **j** is charge current density, *e* the charge of an electron, M_S_ the saturation magnetization of MnGa, and t_F_ the thickness of MnGa^35^. In MnGa/Co/Ta, *P*** **= 0.3, *ρ*_*N*_** **= 125 *μ*Ω*cm*, θ_SH_** **= −0.11, *λ*_*N*_** **= 1.26 *nm, ρ*_*F*_** **= 385 *μ*Ω*cm*, and *t*_*F*_** **= 3.8 *nm* are fixed, and Re [G_MIX_]** **= 10^15^ Ω^−1^cm^−2^ are assumed. *λ*_*F*_ is then calculated to be 2.71 nm. It shows that *λ*_*F*_ is larger than the thickness of the inserted Co layer, indicating less spin scattering at the MnGa/Co interface. For simplicity, in our work, the different parameters for MnGa/Co_2_FeAl/Ta and MnGa/Co/Ta are only *P, λ*_*F*_ and *ρ*_*F*_. Here *P*** **= 0.5, *λ*_*F*_** **= 0.8 *nm* and *ρ*_*F*_** **= 314 *μ*Ω*cm* are assumed, then the SMR of MnGa/Co_2_FeAl/Ta is calculated to be −0.0008%, which is much smaller than its AMR and is hard to be distinguished.

## Discussion

We have designed SOT devices based on MnGa/Ta films after inserting very thin Co_2_FeAl and Co layers to study the modulated switching current density ***J***_**c**_ and SOT. According to the band structures of Co and MnGa, the signs of the spin polarization in Co and MnGa are all negative, leading to the antiferromagnetic exchange coupling at the interface^25^,^36^,^37^,^38^,^39^,^40^,^41^. However, for Co_2_FeAl in both the ordered *L*2_1_ and the partially ordered *B*2 structures, the density of states number at Fermi surface for spin-up bands is larger than spin-down bands, and the spin polarization in Co_2_FeAl is positive^40^,^41^. It is different from MnGa with negative spin polarization and the discontinuity of the band structure at the MnGa/Co_2_FeAl interface becomes more pronounced, leading to a larger Rashba effect. It can also explain the smaller anisotropy and coercivity of MnGa/Co_2_FeAl/Ta, because in this case the interfacial exchange coupling is ferromagnetic^24^. The ferromagnetic coupling in MnGa/Co_2_FeAl has decreased the effective anisotropy field, which is one of the reasons for the decreased switching current density.

Meanwhile, the harmonic measurements have demonstrated that **H**_**F**_ was enhanced in MnGa/Co_2_FeAl/Ta, which also contributes to the smaller ***J***_**c**_. On the contrary, as mentioned above, the effective anisotropy field of MnGa/Co/Ta is larger, which makes it hard to switch the magnetization though there is a remarkable enhancement of **H**_**D**_. However, this analysis of the reversal of PMA layers by in-plane currents employs just a simple macrospin picture of magnetic dynamics. This macrospin description is clearly inadequate for providing accurate quantitative understanding of the reversal process. Lee *et al*. have studied the deterministic magnetic reversal of a perpendicularly magnetized Co layer in a Co/MgO/Ta nanosquare driven by SOT from an in-plane current flowing in a Pt under layer^18^. They have found that the reversal occurs through the nucleation of reversed domains much smaller than the device size, followed by a thermally assisted DW depinning process that results in the complete reversal of the entire Co by the DW propagation. The role of the in-plane magnetic field is to turn the in-plane orientation of the magnetic moments within the DWs to have a significant component parallel to the current flow, thereby allowing the torques from the SHE to produce a perpendicular equivalent field that can expand a reversed domain in all lateral directions. Rojas-Sánchez *et al*. have also experimentally investigated the current-induced magnetization reversal in Pt/[Co/Ni]_3_/Al multilayers^42^. They have shown that the nucleation process occurs at the edge of the tracks carrying the charge current due to the Ørsted field. This demonstrates that the critical switching current also depends on the Ørsted field. The study of DW propagation supports the existence of a Néel DW configuration at zero field, due to the Dzyaloshinski-Moriya interaction (DMI) at the Co/Pt interface. An in-plane magnetic field is required to tune the DW center orientation along the current for efficient DW propagation. According to the magnetic properties and SMR measurements, the modulated magnetic coupling, DMI and SHE in MnGa/Co_2_FeAl/Ta and MnGa/Co/Ta should also induce a variation in the domain structures and propagation, resulting in the modulated switching current density.

Furthermore, the harmonic Hall and SMR measurements have shown that the inserted Co layer has mainly enhanced the SHE, while the Co_2_FeAl layer has mainly enhanced the Rashba effect. Recently, Haney *et al*. have developed semiclassical models for electron and spin transport in bilayer nanowires with a ferromagnetic layer and a nonmagnetic layer with strong SOC^43^. They have proved that the damping-like torque is typically derived from the models describing the bulk SHE and the spin transfer torque, and the field-like torque is typically derived from a Rashba model describing interfacial SOC. The SHE and Rashba interaction do not interfere with each other. That is, the interfacial spin-orbit coupling does not significantly modify the torque due to the bulk SHE. At the same time, it leads to additional torque that is closely related to those found in the two-dimensional Rashba model calculations. The inserting ultrathin Co_2_FeAl or Co layers will modify both Rashba effect and SHE. It can explain the enhanced field-like torques in MnGa/Co_2_FeAl/Ta mainly induced by the Rashba effect, and the enhanced damping-like torques are ascribed to the modulated SHE, which is also proved by the SMR measurement.

Lastly, as shown in Fig. 5a, the resistivity of MnGa/Ta is about 200 μΩ.cm. With measuring the resistivity of Co and Co_2_FeAl layers (ρ_Co_** **= 80 μΩcm, ρ_Co2FeAl_ ****= 60 μΩcm) directly deposited on Si/SiO_2_ substrates, the current in the Ta layer is supposed to be shunted by the inserted Co or Co_2_FeAl layers due to their small resistivity. The shunting effect will reduce the generated spin current in the Ta film and reduce the SHE, resulting in the increased switching current density. However, according to the harmonic Hall and SMR measurements, the SHE has been enhanced in MnGa/Co/Ta as compared with that in MnGa/Ta. Therefore, the influence of the shunting effect is smaller than that induced by the modulated SHE with varying DMI and spin mix conductance at the interface.

Although our work provides an opportunity to tune the ***J***_**c**_ in SOT devices based on PMA MnGa, there is still much room for further theoretical and experimental works towards a better understanding of SOT and the ways to reduce the ***J***_**c**_, which is the ultimate goal for technological applications.

## Methods

In the experiment, a 3-nm-thick *L*1_0_-MnGa single-crystalline film is grown on a semi-insulating GaAs (001) substrate by molecular-beam epitaxy. Then, Ta (5), Co_2_FeAl (0.8)/Ta (5), Co (0.8)/Ta (5), Co (0.8)/Al (5) films (all units are in nanometers) are deposited on it by dc magnetron sputtering, respectively. On the other hand, for measuring the resistivity of Co and Co_2_FeAl layers, Co_2_FeAl (0.8)/Ta (5) and Co (0.8)/Ta (5) are also deposited on Si/SiO_2_ substrates, respectively. Photolithography and Ar ion milling are used to pattern Hall bars and the lift-off process is used to form the contact electrodes.

## Additional Information

**How to cite this article**: Meng, K. *et al*. Modulated switching current density and spin-orbit torques in MnGa/Ta films with inserting ferromagnetic layers. *Sci. Rep.* **6**, 38375; doi: 10.1038/srep38375 (2016).

**Publisher's note:** Springer Nature remains neutral with regard to jurisdictional claims in published maps and institutional affiliations.
